# Somatic genetic alterations in a large cohort of pediatric thyroid nodules

**DOI:** 10.1530/EC-19-0069

**Published:** 2019-05-14

**Authors:** Barbora Pekova, Sarka Dvorakova, Vlasta Sykorova, Gabriela Vacinova, Eliska Vaclavikova, Jitka Moravcova, Rami Katra, Petr Vlcek, Pavla Sykorova, Daniela Kodetova, Josef Vcelak, Bela Bendlova

**Affiliations:** 1 Department of Molecular Endocrinology, Institute of Endocrinology, Prague 1, Czech Republic; 2 Department of Ear, Nose and Throat, 2nd Faculty of Medicine, Charles University in Prague and Motol University Hospital, Prague 5, Czech Republic; 3 Department of Nuclear Medicine and Endocrinology, 2nd Faculty of Medicine, Charles University in Prague and Motol University Hospital, Prague 5, Czech Republic; 4 Department of Pathology and Molecular Medicine, 2nd Faculty of Medicine, Charles University in Prague and Motol University Hospital, Prague 5, Czech Republic

**Keywords:** papillary thyroid cancer, pediatric, mutations, benign, next-generation sequencing

## Abstract

There is a rise in the incidence of thyroid nodules in pediatric patients. Most of them are benign tissues, but part of them can cause papillary thyroid cancer (PTC). The aim of this study was to detect the mutations in commonly investigated genes as well as in novel PTC-causing genes in thyroid nodules and to correlate the found mutations with clinical and pathological data. The cohort of 113 pediatric samples consisted of 30 benign lesions and 83 PTCs. DNA from samples was used for next-generation sequencing to identify mutations in the following genes: *HRAS*, *KRAS*, *NRAS*, *BRAF*, *IDH1*, *CHEK2*, *PPM1D*, *EIF1AX*, *EZH1* and for capillary sequencing in case of the *TERT* promoter. RNA was used for real-time PCR to detect *RET/PTC1* and *RET/PTC3* rearrangements. Total detection rate of mutations was 5/30 in benign tissues and 35/83 in PTCs. Mutations in *RAS* genes (*HRAS* G13R, *KRAS* G12D, *KRAS* Q61R, *NRAS* Q61R) were detected in benign lesions and* HRAS* Q61R and *NRAS* Q61K mutations in PTCs. The *RET/PTC* rearrangement was identified in 18/83 of PTCs and was significantly associated with higher frequency of local and distant metastases. The *BRAF* V600E mutation was identified in 15/83 of PTCs and significantly correlated with higher age of patients and classical variant of PTC. Germline variants in the genes *IDH1*, *CHEK2* and *PPM1D* were found. In conclusion, *RET/PTC* rearrangements and *BRAF* mutations were associated with different clinical and histopathological features of pediatric PTC. *RAS* mutations were detected with high frequency in patients with benign nodules; thus, our results suggest that these patients should be followed up intensively.

## Introduction

Thyroid nodules affect around 1% of the pediatric population. The incidence has been steadily increasing over the last decades, by approximately 1.1% per year worldwide (1, 2). Possible reasons for this increase are improvements in medical care including ultrasonography and fine-needle aspiration biopsy, environmental carcinogens, endocrine disruptors, inadequate iodine intake and exposure to radiation (3). Thyroid nodules have benign or malignant character. The risk of malignancy is higher in pediatric nodules compared with adults, 26 vs 7–15% (2, 4).

Pediatric thyroid cancer is a rare disease occurring mostly in females than in males (1). The most common type is papillary thyroid cancer (PTC), which represents 90% or more of pediatric thyroid cancer (4). Medullary (MTC) and follicular thyroid cancer (FTC) are relatively rare. MTC is mostly familial than sporadic in children and adolescents. Poorly differentiated cancer and anaplastic thyroid cancer (ATC) have only been identified in several pediatric patients (5, 6).

PTCs in children and adolescents are more aggressive than adult PTCs according to many studies (7, 8, 9). On the other hand, they have better outcomes, a lower rate of dedifferentiation and a disease-specific mortality lower than 2% (10). Due to many differences between pediatric and adult PTCs, ATA guidelines specifically for children and adolescents were developed (4).

Differences between pediatric and adult PTCs are not only in clinical-pathological features, but also in genetic alterations. Main PTC-activating somatic mutations in the *RAS*, *BRAF* and *TERT* genes and *RET/PTC* rearrangements cause uncontrolled activation of MAPK and PI3K signaling pathways. It was reported that pediatric PTCs harbor more frequently rearrangements and less frequently point mutations than adult PTCs (11, 12). However, published studies have been performed only in a limited number of pediatric patients or on a low number of investigated genes.

In the Thyroid Cancer Genome Atlas (TCGA) project, genetic alterations of 496 PTCs were detected and mutations in the *CHEK2*, *PPM1D* and* EIF1AX* genes were identified as the novel PTC-causing genes. If these genes play a role in pediatric carcinogenesis is still unknown, because only nine pediatric patients were included in the TCGA project (13).

To the other genes that are associated with thyroid nodules, *IDH1* and *EZH1* genes belong. Mutations in the *IDH1* gene are present in many different types of cancer, including PTC. Several variants have been revealed in the conserved part of the *IDH1* gene with an association with follicular variant of PTC (14). In the *EZH1* gene a hotspot Q571R mutation was detected that causes increased proliferation of thyroid cells (15). This mutation was found predominantly in benign thyroid nodules with follicular pattern and without *RAS* mutation (16).

The purpose of this study was to identify main genetic alterations in one of the largest pediatric cohorts of thyroid nodules. This study investigated not only commonly screened genes *HRAS*, *KRAS*, *NRAS*, *BRAF*, *TERT*, but also newly identified genes *IDH1*, *CHEK2*, *PPM1D*, *EIF1AX* and *EZH1*. From fusion genes the most common *RET/PTC1* and *RET/PTC3* rearrangements were tested.

## Material and methods

### Patients and data collection

The cohort consisted of 113 samples from 83 PTCs and 30 benign lesions. Samples were collected from pediatric patients who underwent surgery from 2003 to 2017 at the Department of Ear, Nose and Throat, 2nd Faculty of Medicine, Charles University and Motol University Hospital in Prague. The thyroid tumor samples were histologically evaluated. All the specimens were snap-frozen and stored at −80°C until used for DNA and RNA isolation. Clinical and histopathological data were obtained from clinical and pathological records. Patients or their legal representatives signed an informed consent for genetic studies approved by the Ethics Committee of the Institute of Endocrinology.

### DNA and RNA extraction

DNA and RNA isolations were performed using the AllPrep DNA/RNA/Protein Mini kit (Qiagen) according to the manufacturer’s instructions. The concentration and purity of DNA and RNA was measured using a spectrophotometer (NanoPhotometer P330; Implen GmbH, München, Germany) and a fluorometer (Qubit 2.0; Invitrogen). The RNA integrity was evaluated using a Bioanalyzer 2100 and Agilent RNA 6000 Nano Kit (Agilent Technologies).

### Next-generation sequencing

The analyzed genes were *HRAS* (exons 2, 3), *KRAS* (exons 2, 3), *NRAS* (exons 2, 3), *BRAF* (exon 15), *TERT* (promoter), *IDH1* gene (exons 4, 6), *CHEK2* (exons 3, 4, 7, 11, 13), *PPM1D* (exons 1, 4, 5, 6), *EIF1AX* (exons 1, 2, 5, 6), *EZH1* (exons 16, 17). Exons of *RAS*, *BRAF*, *CHEK2*, *PPM1D*, *EIF1AX* genes and the *TERT* promoter were selected for analysis because they were the most mutated regions in the TCGA study (13). Exons of *IDH1* and *EZH1* genes were chosen according to previous publications (14, 16). Exons were amplified using PCR with sequence-specific primers. The primer sequences and PCR conditions are available on request. The PCR products were purified using Agencourt AMPure (Beckman Coulter) and used for preparation of next-generation sequencing (NGS) libraries using Nextera XT Sequencing Kit (Illumina) according to the manufacturer’s sample preparation protocol and modifications as described in our previous article (17). The NGS libraries were paired-end sequenced for 500 cycles by MiSeq Reagent kit v2 (Illumina) using MiSeq sequencer platform (Illumina).

All variants of the novel PTC-causing genes from TCGA study were also analyzed in DNA samples extracted from peripheral blood of the same patient to distinguish somatic and germline detected genetic changes.

The reference sequences of genes *HRAS*, *KRAS*, *NRAS*, *BRAF*, *IDH1*, *CHEK2* and *PPM1D* were NM_005343.3, NM_033360.3, NM_002524.4, NM_004333.4, NM_005896.3, NM_007194.3 and NM_003620.3, respectively.

### Capillary sequencing

The PCR products of *TERT* promoter were purified using Agencourt AMPure (Beckman Coulter), and then sequencing reaction using Quick Start Master Mix Kit with Dye Terminator Cycle Sequencing (Beckman Coulter) was performed. Products of the sequencing reaction were purified using Agencourt Clean SEQ Dye-Terminator Removal (Beckman Coulter). Capillary sequencing was performed by CEQ 8000 instrument (Beckman Coulter). Analyses were evaluated by CEQ 8000 software. The primer sequences and PCR conditions are available on request.

### Real-time PCR

RNA was reverse transcribed into cDNA using random primers and AMV Reverse Transcriptase (Promega). Subsequently, cDNA was mixed with iQ SYBR Green Supermix (Bio-Rad) and sequence-specific primers and *RET/PTC1* and *RET/PTC3* detection was performed by Real-Time PCR (Light Cycler 480, Roche). Reaction conditions and primers were set according to our previous article (18). For cDNA quality control, the expression of housekeeping gene (β-actin, *ACTB*) was tested in every sample. Analyses were evaluated by Light Cycler® 480 SW 1.5.1.

### Statistical analysis

Statistical analysis was performed with *t*-test and Fisher’s exact test. *P* value <0.05 was considered as statistically significant.

## Results

### Patient characteristics

The study consisted of 113 specimens of pediatric patients with predominance of females (73.5%). The age ranged 6–20 years at the time of diagnosis.

**Figure 1:**
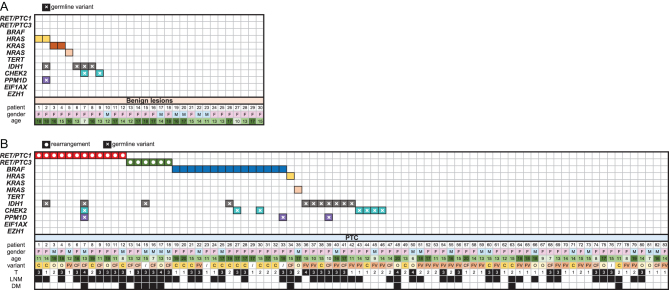
Tile plot of genetic alterations detected in patients with benign lesions (A) and with PTC (B). Clinical and pathological data as gender, age at diagnosis, histological variant, tumor stage, lymph node metastases and distant metastases are shown. C, classical variant; CF, classical and follicular variant; DM distant metastases; F, female; FV, follicular variant; LNM, lymph node metastases; M, male; O, other variant; PTC, papillary thyroid cancer; T, tumor size and extension.

As detailed in Fig. 1 and Table 1, 30 samples of benign lesions were collected (nine solitary thyroid nodules, eight follicular adenomas, five multinodular goiters, five chronic lymphocytic thyroiditis, two thyroid cysts, one oncocytic adenoma). The cohort of benign patients had a mean age at diagnosis 14.7 ± 2.6 years and most of the group consisted of females (24/30). The half of the patients underwent hemithyroidectomy and the other half total thyroidectomy, and in two of them the lymph node dissection was performed. Multifocal nodules in six cases were noted.

**Table 1:** Characteristics of pediatric benign cohort.

	Pediatric benign cohort (*n* = 30)
Patients	
Females	24
Males	6
Age at diagnosis (years, mean ± s.d.)	14.7 ± 2.6
Type of disease	
Solitary thyroid nodule	9
Follicular adenoma	8
Multinodular goiter	5
Chronic lymphocytic thyroiditis	5
Thyroid cyst	2
Oncocytic adenoma	1
Surgery	
Hemithyroidectomy – left lobe	8
Hemithyroidectomy – right lobe	7
Total thyroidectomy without LND	13
Total thyroidectomy with LND	2
Tissue characteristics	
Mean ± s.d. (mm)	22.2 ± 12.1
Multifocality	6

LND, lymph node dissection.

Medical records of 83 patients with PTCs were studied for family history, another serious disease and radiation exposure before PTC diagnosis. Thyroid disease in family history had 25 patients. Except two, no other patient had thyroid cancer in family. Two patients had known previous history of radiation exposure. The first one was diagnosed with acute lymphoblastic leukemia and the therapeutic whole-body radio-exposition was prior to thyroid cancer diagnosis. The second patient was diagnosed with Hodgkin’s lymphoma and was treated with chemotherapy and radiotherapy. One patient was diagnosed with Cowden syndrome, which is associated with a higher risk of thyroid cancer due to mutation in the *PTEN* gene.

Detailed patient’s characteristics of the PTC cohort are summarized in Fig. 1 and Table 2. PTC patients were predominantly females (59/83). Total thyroidectomy was performed in 72 PTC cases and hemithyroidectomy in 11 PTC cases. Hemithyroidectomy was completed to total thyroidectomy in ten cases due to diagnosis of PTC. Mean size of tumor was 22.2 ± 13.6 mm. Microcarcinomas defined as tumors with diameter 10 mm or smaller were detected in 16/83 cases. The most common variant of PTC was follicular variant followed by the classical variant. Many rare variants such as columnar, clear cell, tall cell and diffuse sclerosing variant were identified. More than half PTCs were multifocal (46/83) and nearly half had extrathyroidal extension (37/83) and were classified as T3/T4 (38/83). Lymph node metastases were detected in 52/83 and distant metastases in 10/83 cases. All distant metastases were affecting lungs. Most of the PTC patients (72/82) underwent radioactive iodine (RAI) treatment. One 7-year-old patient died from the disease after surgery and before RAI treatment. Maximum number of RAI ablations was 6, which was used in two cases. The RAI treatment was applied in the first case during 9 years and in the second case during 11 years. In the first case, it was PTC with mix of classical, follicular and solid variant and in the second case, it was solid variant of PTC. Reoperations of lymph node metastases were performed in five patients; two of them underwent reoperations three times.

**Table 2:** Characteristics of pediatric PTC cohort.

	Pediatric PTC (*n* = 83)
Patients	
Females	59
Males	24
Age at diagnosis (years, mean ± s.d.)	14.2 ± 3.4
History of radioexposure	2
Tumor size	
Mean ± s.d. (mm)	22.2 ± 13.6
Microcarcinoma (≤10 mm)	16
Histological variant^a^	
Classical	23
Classical and follicular	16
Follicular	27
Solid	3
Classical, follicular and solid	3
Diffuse sclerosing	2
Columnar	2
Tall cell	1
Clear cell	1
Pathological characteristics	
Multifocality	46
Extrathyroidal extension	37
Intravascular invasion	22
T1/T2 classification	45
T3/T4 classification	38
Lymph node metastases (N)	52
Distant metastases (M)	10
Chronic lymphocytic thyroiditis	42
Radioiodine therapy^b^	
Without radioiodine therapy	9
One dose	41
Two or more doses	31
Follow-up^c^	
Reoperation of recurrent metastases	5
Remission	49
Recurrence or persistence	9
Biochemical persistence	16
Disease-specific mortality	1

^a^In five cases histological variant was not available; ^b^in two cases radioiodine therapy records were not available; ^c^eight cases were not classified due to short-term follow-up.

PTC, papillary thyroid cancer.

Median follow-up was 72 months (range 1–170 months). Thyreoglobulin (Tg) blood serum level and thyreoglobulin antibodies (a-Tg) were monitored for whole postoperative care. Remission was defined as a condition of the patient with any suspect object on ultrasound or whole-body scintigraphy, the Tg levels were lower than 1 µg/L and the a-Tg levels were not detectable. Recurrence and persistence were defined as a condition of a patient with malignant object or objects on ultrasound or whole-body scintigraphy at least 1 year after surgery or patient who entered remission and then malignant object was appeared. Biochemical persistence was defined as a condition of the patient with the level of Tg higher than 1 µg/L or detectable level of a-Tg with no evident tumor recurrence or persistence. Eight patients could not be classified due to short-term follow-up (range 0–12 months after surgery).

### Mutational analysis

The detection rate of mutations in benign and malignant cohorts is noted in Table 3. The total detection rate of mutations in the cohort of benign tissues was 5/30 and in the cohort of PTCs was 35/83, of which *RET/PTC* rearrangement (18/83), *BRAF* V600E mutation (15/83) and *RAS* mutations (2/83) were identified (Fig. 1). All mutations in benign cohort were in *RAS* genes (Fig. 1). Mutations of *RAS* genes in benign lesions included *HRAS* G13R mutation found in one follicular adenoma with uncertain behavior and in one solitary thyroid nodule with oncocytic changes, *KRAS* G12D mutation in the multinodular goiter, *KRAS* Q61R mutation in the follicular adenoma and *NRAS* Q61R mutation in the multinodular goiter with oncocytic changes. Only two mutations in *RAS* genes were identified in PTCs: *HRAS* Q61R and *NRAS* Q61K. *NRAS* Q61K mutation was found in tall cell variant of PTC and *HRAS* Q61R mutation in the mix of classical and follicular variant of PTC. Both patients with *RAS* mutation had lymph node metastases and multifocal carcinoma. The patient with *HRAS* mutation was diagnosed with Hodgkin’s lymphoma three years prior to diagnosis of PTC. The tumor had very invasive character; intravascular invasion and lung metastases were detected.

**Table 3:** Comparison of detection rate of mutations in benign and malignant nodules.

Mutation	Benign (*n* = 30)	Malignant (*n* = 83)	*P* value
*HRAS* G13R	2	0	
*HRAS* Q61R	0	1	
*KRAS* G12D	1	0	
*KRAS* Q61R	1	0	
*NRAS* Q61K	0	1	
*NRAS* Q61R	1	0	
***RAS* total**	5	2	**0.014**
***BRAF* V600E**	0	15	**0.010**
***TERT***	NA	0	
*RET/PTC1*	0	11	
*RET/RET1ex9*	0	1	
*RET/PTC3*	0	6	
***RET/PTC* total**	0	18	**0.003**
**Total**	5	35	**0.014**

NA, not analyzed. Bold indicates statistical significance.

Clinical-pathological features of *RET/PTC* and *BRAF*-positive patients are summarized and compared in Table 4. The most common mutation was the *RET/PTC* rearrangement detected in 18/83 PTCs, including *RET/PTC1* in 11 and *RET/PTC3* in 6 samples. In single case, novel *RET/PTC* rearrangement named *RET/PTC1ex9*, which included part of exon 9 of the *RET* gene, was also identified (18). The ratio between females and males in *RET/PTC*-positive patients was lower compared to *BRAF* positive patients, but not statistically significant (*P* = 0.070). Samples with *RET/PTC* rearrangement were significantly associated with mix of classical and follicular variant of PTC (*P* = 0.009) and higher frequency of lymph node metastases (*P* = 0.020) and distant metastases (*P* = 0.005). All six *RET/PTC3*-positive patients had lymph node metastases, four of these six patients had lung metastases and one patient died from disease. The *BRAF* V600E mutation was detected in 15/83 PTC samples. In *BRAF* positive patients the mean age at the time of diagnosis was significantly higher than in *RET/PTC* positive patients (*P* = 0.012). The *BRAF* mutation also correlates with the classical variant of PTC (*P* < 0.001). The number of patients, who underwent reoperation of recurrent lymph node metastases, was higher, but not significantly (*P* = 0.073). Five patients did not respond to RAI treatment because they did not accumulate RAI. Mutations in the *BRAF* gene and *RET/PTC* rearrangements were exclusively found in malignant samples.

**Table 4:** The comparison of clinical and pathological features between *RET/PTC*- and *BRAF*-positive patients.

	*RET/PTC (n = 18)*	*BRAF (n = 15)*	*P* value
Patients			
Females/males	10/8 (1.25:1)	13/2 (6.5:1)	0.070
Age at diagnosis (mean ± s.d.)	13.4 ± 3.6	16.3 ± 2.4	**0.012**
Tumor size			
Mean ± s.d. (mm)	29.6 ± 18	20 ± 10.6	0.079
Microcarcinoma (≤10 mm)	3	4	0.674
Histological variant^a^			
Classical	4	11	**<0.001**
Classical and follicular	8	0	**0.009**
Follicular	1	1	1.000
Other	4	0	0.121
Pathological characteristics			
Multifocality	11	8	0.733
Extrathyroidal extension	12	6	0.170
Intravascular invasion	7	3	0.283
T1/T2 classification	5	9	0.085
T3/T4 classification	13	6	
Lymph node metastases (N)	16	7	**0.020**
Distant metastases (M)	6	0	**0.005**
Chronic lymphocytic thyroiditis	9	4	0.284
Radioiodine therapy^b^			
Without radioiodine therapy	2	2	1.000
One dose	8	5	0.725
Two or more doses	8	7	1.000
Follow-up^c^			
Reoperation of recurrent metastases	0	3	0.073
Remission	10	6	0.285
Recurrence or persistence	1	3	0.295
Biochemical persistence	6	5	1.000
Disease-specific mortality	1	0	1.000

^a^In three *BRAF*-positive cases and in one *RET/PTC* positive case histological variant was not available; ^b^in one *BRAF*-positive case radioiodine therapy record was not available; ^c^one *BRAF*-positive case was not classified due to short-term follow-up.

Bold indicates statistical significance.

The* TERT* promoter was analyzed only in malignant samples and no mutation was found.

Detected variants in the *IDH1*, *CHEK2* and* PPM1D* genes are summarized in Fig. 1 and Table 5. Variants G105G and V178I in the *IDH1* gene always co-occurred and were detected with comparable frequency 9/83 in malignant and 3/30 in benign samples. The third genetic variant in the *IDH1* gene was Y183C. In the *CHEK2* gene R117G substitution and T367Mfs*15 deletion was identified only in PTC samples. I157T variant was the most common variant in the *CHEK2* gene and was found predominantly in malignant samples. L467F variant was detected only in one patient with follicular adenoma. In the *PPM1D* gene A152A variant was identified only in PTCs and I496V variant was detected in the benign solitary node together with variants of *HRAS* G13R and three variants G105G, V178I and Y183C in the *IDH1* gene. The differences between malignant and benign cohorts were not statistically significant (Table 5). All variants identified in *IDH1*, *CHEK2* and* PPM1D* genes were also tested in DNA from peripheral blood of the same patients that we had tissue and we revealed all variants as germline. No variants in genes *EIF1AX* and *EZH1* were identified in malignant or in benign samples.

**Table 5:** Identified variants in *IDH1*, *CHEK2* and *PPM1D* genes.

Gene	Exon	Amino acid	Nucleotide	SNP ID	Clinical significance (ClinVar)	SIFT	PolyPhen	Patients with benign tissues (*n* = 30)	Patients with PTCs (*n* = 83)	*P* value	Frequency of altered allele in population (GnomAD, %)
*IDH1*	4	G105G	c.315C>T	rs11554137	Benign	–	–	3	9	1	5.050
	6	V178I	c.532G>A	rs34218846	Benign	Tolerated	Benign	3	9	1	4.861
	6	Y183C	c.548A>G	rs34599179	Untested	Deleterious	Probably damaging	2	2	0.287	0.986
*CHEK2*	3	R117G	c.349A>G	rs28909982	Likely pathogenic	Deleterious	Probably damaging	0	1	1	0.012
	4	I157T	c.470T>C	rs17879961	Risk factor^a^	Tolerated	Benign	1	5	1	0.426
	11	T367Mfs*15	c.1100delC	rs555607708	Pathogenic	–	–	0	1	1	0.208
	13	L467F	c.1401G>C	rs876658908	Uncertain significance	Deleterious	Probably damaging	1	0	0.265	NA
*PPM1D*	1	A152A	c.456C>T	rs149400522	Benign	–	–	0	3	0.564	0.444
	6	I496V	c.1486A>G	rs35491690	Likely benign	Tolerated	Benign	1	0	0.265	0.081

^a^Conflicting interpretations of pathogenicity (uncertain significance/likely pathogenic/pathogenic).

GnomAD, Genome Aggregation Database; NA, not available; PTC, papillary thyroid cancer; SIFT, Scale Invariant Feature Transform; SNP, single nucleotide polymorphism.

## Discussion

Thanks to the development of NGS and using this technique in molecular genetics laboratories, it is possible to study the precise and comprehensive genetic landscape of pediatric thyroid nodules. It helps not only to increase detection rate, but also to identify new molecular markers or cancer gene predispositions. Several years ago, only small cohorts of pediatric patients and few selected numbers of genetic alterations were investigated. Recently, studies with larger cohorts of pediatric patients have appeared with enlarging the spectrum of investigated genetic mutations using NGS panels to find mutations or gene fusions (12, 19, 20). Our study with the size of 113 samples of thyroid nodules is according to our best knowledge the largest pediatric cohort that was analyzed by NGS and provides very important genetic, clinical and pathological data. Unlike our study, in previous studies where NGS panels were also used, presented a limited number of pediatric PTC specimens that ranged from 13 to 25 (12, 19, 20). On the other hand, NGS panels included more genes (14–250) in these studies (12, 19, 20). Moreover, mutations in genes *TET*, *TSHR* (12) and *CDKN2A* (19) and rearrangements of *ALK*, *NTRK1*, *NTRK3*, *PPARγ* genes were revealed (12, 20), besides point mutations in the genes identified also in our study.

Mutations in *RAS* genes occur in malignant and benign pediatric thyroid nodules overall with very low frequency (20, 21, 22, 23). In our cohort, *NRAS* and *HRAS* mutations were detected with no other co-mutation in PTCs with aggressive character, and surprisingly, five *RAS*-positive samples were identified in benign lesions. Clinical impact and prognostic importance of *RAS* mutations is not clearly established, because they appear in follicular adenoma and also in ATC. The risk of malignancy varies among *RAS* genes (*HRAS* 92%, *NRAS* 74%, *KRAS* 61%) in adult population (24). When we consider that pediatric patients have overall higher risk of malignancy than adults, the numbers could be even higher. In our benign cohort, oncocytic changes or uncertain behaviour were identified in HRAS and NRAS-positive samples, but not in KRAS-positive samples. *RAS* mutations could play a role in early cancerogenesis (25). It implies that the pediatric patients with benign lesions harboring *RAS* mutations should be more intensively followed.

The *RET/PTC* rearrangement was the most frequent mutation in our cohort of pediatric PTCs, detected in 18/83 malignant samples exclusively. The prevalence of *RET/PTC* in pediatric PTCs is worldwide in the range of 15–67% (7, 8, 26, 27). However, these fusion genes were also found in benign pediatric samples (28). *RET/PTC* is commonly associated with radiation-induced PTC, in which the prevalence is higher (7). In our study, all *RET/PTC*-positive patients were without radiation history. In literature, *RET/PTC* rearrangements do not harbor in general other driver co-mutation in the *BRAF* or *RAS* genes, which was also confirmed in our study. This co-occurrence was found only in few cases and it was associated with more aggressive disease (8, 29). *RET/PTC*-positive samples were associated (not statistically significant) with a higher rate of tumor growth (mostly T3/T4 classification), more frequent extrathyroidal extension, intravascular invasion and multifocality compared to *BRAF*-positive samples. They were significantly associated with more frequent local and distant metastases, especially in cases of *RET/PTC3* rearrangement. No patient underwent reoperation due to recurrent metastases.

The *BRAF* V600E mutation was completely associated with PTC. In our cohort of pediatric PTCs, the prevalence of the *BRAF* V600E was 15/83, which falls within the range of 0–63% of the prevalence *BRAF* mutation in pediatric cases published earlier (7, 9, 30, 31). Possible reasons for wide dispersal of prevalence are different used methods, geographical and environmental factors, size of cohort and age border of pediatric patients. Our study also confirmed that the *BRAF* mutation correlates with the classical variant of PTC and the higher age of patients (32). The association of the *BRAF* V600E mutation with more frequent T1/T2 classification than T3/T4 classification and with higher rate of recurrence was seen, but was not statistically significant (Table 4). *BRAF* V600E-positive patients had more lymph node metastases reoperations due to radiorefracterity than *RET/PTC*-positive patients, but the difference was not statistically significant.

In the *IDH1* gene, the germline variants G105G and V178I in similar frequencies in patients with benign and malignant nodules were found. Clinical significance of these variants seems according to ClinVar database and SIFT/PolyPhen *in silico* analysis benign on thyroid tumorigenesis. The Y183C variant was detected with higher frequency in benign nodules. However, this variant was assumed as deleterious/probably damaging by SIFT and PolyPhen analysis. Clinical effect of Y183C variant is still unknown. None of the previously described variants around codon R132 was found, where the active site of the enzyme occurs (14).

In the TCGA study PTC-causing somatic mutations in *CHEK2*, *PPM1D* and *EIF1AX* genes were identified. Our study revealed several variants in *CHEK2* and *PPM1D* genes that were detected in benign as well as in malignant nodules. These variants did not correspond to those found in the TCGA study (13). All our detected variants in the *CHEK2* and *PPM1D* genes were germline. I157T variant in the *CHEK2* gene was detected in one benign tissue and in five PTCs, which falls in the worldwide range 4.5–15.6% of PTC (33, 34, 35). It was reported that this variant increases the risk of PTC almost twice (OR = 1.81) (35). In PTCs, also other variants R117G and T367Mfs*15 in the *CHEK2* gene with probably deleterious effect on protein function were detected (36). It was published that truncating variants are associated with higher risk of thyroid cancer (OR = 5.7) than I157T missense variants (OR = 2.8) (34). In the *PPM1D* gene two variants were detected in our cohorts with probably benign significance on thyroid tumorigenesis. In our study, any variant in the *EIF1AX* gene was revealed. Frequency of mutations in this gene is generally very low and we assume according to our results, that there is probably no correlation between pediatric thyroid nodules and *EIF1AX* mutations. In one recent study, these novel genes were investigated in pediatric and adult cohorts with PTCs. In pediatric patients, they detected five variants in the *CHEK2* gene, which were presented in all samples, two variants in the *PPM1D* gene and none in the *EIF1AX* gene. Found variants in the *PPM1D* gene did not match ours (37).

Any mutation in the *TERT* promoter in pediatric PTCs was detected. Mutations in this gene are strongly associated with the higher age of adult patients (38). In many studies of pediatric PTC, mutation in the *TERT* promoter has not yet been detected all over the world (9, 19), except one study. They found C228T mutation in 10-year-old patient without extrathyroidal invasion and metastases (39).

In the *EZH1* gene, no mutation was found in benign and malignant specimens in our cohorts. The reason was probably a small cohort of benign samples, because mutations in the *EZH1* gene were detected almost 20 times more frequently (13.5 vs 0.7%) in benign than in malignant thyroid nodules (16). Mutations in the *EZH1* gene were identified only in two PTC cases in the TCGA study (13).

In summary, we revealed genetic cause in 5/30 of benign nodules and in 35/83 of pediatric PTCs; mutations in the *BRAF* gene, *RAS* genes and *RET/PTC* rearrangements were included. There were significant differences in clinical courses in *RET/PTC* and *BRAF-*positive PTC patients. Patients with *RET/PTC* had more aggressive disease (more frequent local and distant metastases) than patients with *BRAF* mutation. On the basis of the known genetic change in tumor, it could be possible to stratify and individualize the treatment. The role of *RAS* mutations, especially in benign lesions, is not yet fully understood, so it should be kept in mind that they may predispose to cancer and the intensive follow-up of these patients is recommended. Some of the identified variants in the *CHEK2* gene are pathogenic or likely pathogenic and the influence of found variants in the *IDH1* and *PPM1D* gene on thyroid nodules is still uncertain and probably benign.

The genetic landscape of more than half of our PTC cases is still unknown; therefore, further investigation is required, for example in *PTEN* or *PIK3CA* genes, in which somatic mutations in pediatric PTCs were detected (22). Furthermore, other fusion genes need to be identified by RNA sequencing, and copy number variants should also be detected, which could be another trigger of thyroid cancer. The genetic molecular testing seems to be the benefit for pediatric patients for their diagnosis and prognosis. Hopefully, it will improve the quality of life of pediatric patients with thyroid nodules.

## Declaration of interest

The authors declare that there is no conflict of interest that could be perceived as prejudicing the impartiality of the research reported.

## Funding

This work was supported by the Ministry of Health of the Czech Republic AZV (16-32665A) grants.
